# Arterial stiffness in patients with type 1 diabetes and its comparison to cardiovascular risk evaluation tools

**DOI:** 10.1186/s12933-022-01537-1

**Published:** 2022-06-09

**Authors:** Simon Helleputte, Luc Van Bortel, Francis Verbeke, Jos Op ‘t Roodt, Patrick Calders, Bruno Lapauw, Tine De Backer

**Affiliations:** 1 Faculty of Medicine and Health Sciences, Ghent University, Corneel Heymanslaan 10, 9000 Ghent, Belgium; 2 Fonds Wetenschappelijk Onderzoek (FWO) Vlaanderen, Ghent, Belgium; 3 Unit of Clinical Pharmacology, Department of Basic and Applied Medical Sciences, Ghent University, Ghent, Belgium; 4 Department of Nephrology, Ghent University Hospital, Ghent, Belgium; 5 Maastricht University Medical Centre (MUMC), Maastricht, The Netherlands; 6 Department of Endocrinology, Ghent University Hospital, Ghent, Belgium; 7 Department of Cardiology, Ghent University Hospital, Ghent, Belgium

**Keywords:** Arterial Stiffness, Cardiovascular Risk, ESC guidelines, STENO risk engine, Type 1 Diabetes, Pulse Wave Velocity

## Abstract

**Background:**

Arterial stiffness is a potential biomarker for cardiovascular disease (CVD) risk in patients with type 1 diabetes (T1D). However, its relation with other CV risk evaluation tools in T1D has not been elucidated yet. This study aimed to evaluate arterial stiffness in T1D patients free from known CVD, and compare it to other CV risk evaluation tools used in T1D.

**Methods:**

Cross-sectional study in adults with a T1D duration of at least 10 years and without established CVD. Patients were categorized in CVD risk groups based on *2019 European Society of Cardiology (ESC) guidelines*, and the STENO T1D risk engine was used to estimate 10-year risk for CV events. Arterial stiffness was evaluated with carotid-femoral pulse wave velocity (cf-PWV). Coronary artery calcium (CAC) score was assessed and carotid ultrasound was performed. Ambulatory 24-h blood pressure and central hemodynamic parameters were evaluated. Data on renal function and diabetic kidney disease was retrieved.

**Results:**

54 patients (age: 46 ± 9.5 years; T1D duration: 27 ± 8.8 years) were included. One-fourth of patients showed prematurely increased aortic stiffness based on cf-PWV (24%). Cf-PWV was significantly associated with CAC score, carotid intima-media thickness, central hemodynamic parameters and diabetic kidney disease. Based on STENO, 20 patients (37%) were at low, 20 patients (37%) at moderate, and 14 patients (26%) at high 10-year risk for CV event. Cf-PWV was strongly associated with the STENO score (r_s_ = + 0.81; R^2^ = 0.566, p < 0.001), increasing with each higher STENO group (p < 0.01). However, cf-PWV was not significantly different between the two CV risk groups (high versus very high) based on ESC criteria, and ESC criteria compared to STENO classified 10 patients more as having > 10% 10-year risk for CV events (n = 44/54; 81.5% versus n = 34/54; 63%).

**Conclusions:**

This study demonstrated that a substantial proportion of long-standing T1D patients free from known CVD show premature arterial stiffening. Cf-PWV strongly associates with the STENO risk score for future CV events and with cardiovascular imaging and function outcomes, thereby illustrating the clinical importance of arterial stiffness. The data, however, also show considerable heterogeneity in CV risk and differences in risk categorisation between the STENO tool and ESC criteria.There is a need for refinement of CV risk classification in T1D, and future studies should investigate if evaluation of arterial stiffness should be implemented in T1D clinical practice and which patients benefit the most from its assessment.

## Background

Despite substantial improvements in patient care and significant reductions in cardiovascular disease (CVD) morbidity and mortality already achieved in the past decade [1–3], patients with type 1 diabetes mellitus (T1D) are still characterized by an increased cardiovascular burden [2, 4, 5]. According to the latest *European Society of Cardiology (ESC) guidelines on diabetes, pre-diabetes and CVD*, patients with a longer T1D duration (i.e., > 10 years) are automatically considered at high- or very high risk to develop CVD [6]. However, there seems to be substantial heterogeneity in CV risk in T1D [7, 8]. Therefore, it is crucial to accurately assess CV risk in patients with T1D and there is growing research interest into CV risk stratification tools [8–11].

The fact that some T1D patients develop CVD despite adequate control of traditional CV risk factors or vice versa, illustrates that total CV risk is not entirely captured by these factors alone [3, 12]. Since routine CVD screening in asymptomatic T1D patients remains currently not recommended [13], there is a need for alternative biomarkers for CV risk [3, 14, 15], of which arterial stiffness is of particular interest. Arterial stiffness is considered an ‘*integrative measure*’ of all CV risk markers on the arterial wall [16, 17], *i.e.* taking into account also other deleterious processes that contribute to increased CV risk such as low-grade inflammation or oxidative stress [12]. Arterial stiffness was found to be increased in patients with T1D compared to healthy age-matched controls independent of traditional CV risk factors [18–21]. More importantly, carotid-femoral pulse wave velocity (cf-PWV)—the gold-standard measure for arterial stiffness [22]—showed important independent prognostic value in the development of CVD and mortality in both healthy and diseased populations [23–25]. In patients with T1D, aortic PWV was associated with the presence of micro- and macrovascular disease [26] and a more recent prospective study suggested a predictive role of cf-PWV for CV events and mortality [27].

However, the question remains how cf-PWV compares to other CV risk evaluation methods in asymptomatic patients with T1D. A T1D-specific CV risk assessment tool is the STENO engine, a validated prediction model developed to estimate 10-years risk for CV events in patients with T1D without previous CVD [10]. On the other hand, CV risk classification in T1D can also be based on the ESC guidelines [6].

The current study aimed to evaluate arterial stiffness assessed by cf-PWV in patients with a T1D duration of at least 10 years and still free from known CVD, and to compare cf-PWV to other CV risk evaluation tools used in T1D.

## Methods

### Study design and subjects

Patients with T1D took part in this cross-sectional study as part of a larger CV screening program at our tertiary care centre (*Ghent University Hospital, Belgium*). Inclusion criteria were: age ≥ 18 years, T1D duration > 10 years and absence of known CVD (*i.e*., no history of angina pectoris, acute coronary syndrome, cerebrovascular accident/stroke, symptomatic peripheral artery disease or any CV procedure). The study was approved by the local Ethics Committee and all patients provided written informed consent.

### Measurements

#### Patient and disease characteristics

HbA1c was determined with high-performance liquid chromatography (HPLC) (*Automated Glycohemoglobin Analyzer HLC-723 G8; Tosoh® Bioscience Company, Tokyo, Japan*) on a fasting blood sample. Lipid profiles (LDL-, HDL-, total cholesterol, and triglycerides (TGL)) were determined. LDL-C values were evaluated according to both the 2016 as well as the latest 2019 *ESC/EAS Guidelines for the management of dyslipidaemias*, with target values being < 100 or < 70 mg/dl for patients with T1D > 10 or > 20 years, respectively, according to 2016 guidelines [28]; or < 70 or < 55 mg/dl, respectively, according to the 2019 guidelines [29]. Information on antihypertensive and lipid-lowering treatment was collected.

Ambulatory blood pressure monitoring (ABPM) was performed, with brachial BP being recorded for 24 h at the non-dominant arm (*Spacelabs Healthcare® 90217A; Issaquah, Washington, USA*), every 15 or 30 min during day- or night-time (12AM-6AM), respectively. Patients were instructed to perform their normal daily activities. SBP, DBP, MBP for daytime, night-time and 24 h were collected, and definitions of hypertension were based on European Society of Hypertension (ESH) guidelines [30]. SBP dipping (= (daytime – night-time SBP) / daytime SBP × 100) was reported, with < 10% considered as non-dipping.

Data on renal function, serum creatinine, estimated GFR (eGFR; CKD-EPI equation) and presence of albuminuria on 24-h urine collection (urine albumin-to-creatinine ratio (UACR)) were collected; with diabetic kidney disease being defined as albuminuria (UACR ≥ 30 mg/g creatinine or 30 mg per 24 h), or use of a RAAS-inhibitor for albuminuria, or eGFR < 60 mL/min/1.73m^2^. Information on the presence of retinopathy was retrieved through consultation of patients’ electronic medical records.

#### Cardiovascular risk evaluation

*STENO risk engine*. This validated T1D-specific tool (*Steno Diabetes Center, Copenhague*) [10] was used to estimate 10-year risk for future CV events (fatal and non-fatal) in T1D patients without previous CVD. The prediction model is based on ten variables (age, gender, T1D duration, smoking, SBP, LDL-C, HbA1c, eGFR, albuminuria, and physical activity level), and patients are categorized into low (< 10%), medium (10.0–19.9%) or high CV event risk (≥ 20%).

*ESC 2019 guidelines on diabetes and CVD.* Based on these guidelines, patients can be classified into two categories for 10-year risk of fatal CVD: (1) very high risk (≥ 10%), including patients with early-onset T1D of long duration (> 20 years), or target organ damage, or three or more major CV risk factors; and (2) high risk (5–10%), comprising all patients not included in the first category [6].

#### Arterial stiffness: Carotid-femoral pulse wave velocity (cf-PWV)

Stiffness of the aortic segment was calculated as the travel distance between the carotid and femoral artery location, divided by the difference in transit time of the pulse wave between the heart (based on R-top identification on ECG) and these two locations [22, 31]. Measurements were performed with the SphygmoCor device (*AtCor Medical®, Sydney, Australia*) and according to consensus guidelines [22]. All patients were evaluated at the same time of day (8AM) to minimize influence of diurnal variation in blood vessel tone, after 8-h overnight fasting, and abstained from vasoactive medication, caffeine, tea, polyphenol-rich foods, alcohol, nicotine and strenuous exercise in the 24 h prior to testing. Glycaemia was monitored and measurements were only performed if glycaemia was in the range of 70–250 mg/dl, *i.e.* no hypoglycaemia or extreme hyperglycaemia. Measurements were performed in a quiet room after ten minutes of baseline rest, at the right body side, with patients in supine position and not allowed to speak or sleep. Common carotid and femoral artery pulse waves were directly measured with applanation tonometry, with the time delay in pulse wave arrival determined according to the foot-to-foot method. The direct carotid-femoral travel distance was measured with an infantometer after precise determination of the arteries’ pulse location and 80% of the direct distance was used in cf-PWV calculation [22, 31]. A measurement was only accepted if the quality criteria indicated by the device were met. Measurements were performed at least twice with a maximum difference of 0.5 m/s between measurements allowed, otherwise a third measurement was executed. The mean or median value of these two or three measurements, respectively, was used. Obtained cf-PWV values were compared to the *Reference Values for Arterial Stiffness' Collaboration* [32] – providing reference values in > 11,000 non-diabetic European people free from overt CVD – to determine whether increased arterial stiffness was present, *i.e.,* cf-PWV above the 90^th^ percentile of the age- and BP-matched reference value. The absolute cut-off of > 10 m/s (expert consensus) for *increased CV event risk* was also applied [22].

#### Cardiovascular imaging and function outcomes

*Coronary artery calcium (CAC) score.* Computed tomography (CT) scan was used to non-invasively assess atherosclerotic burden by evaluating coronary artery calcifications, expressed in Agatston units. Patients were categorized based on absolute values as were used in DCCT/EDIC analyses [33], with CAC score 0: no calcifications; 1–99: mild calcifications; 100–299: moderate calcifications; ≥ 300: severe calcifications. Age- and sex-adjusted percentiles [34] were also used for categorisation, with < P_50_: normal calcification; P_50-75_: mild calcifications; P_75-90_: moderate calcifications; and > P_90_: severe calcifications.

*Carotid duplex ultrasound.* B-mode and colour Doppler ultrasound (*GE Healthcare® Vivid E95*) was used to evaluate carotid intima-media thickness (IMT), presence of carotid plaques and stenosis.

*Central hemodynamics.* Applanation tonometry of the radial artery was used for estimation of central BP parameters. First, brachial office BP was measured three times (*Omron® IntelliSense 705IT*) with two minutes in between, and the mean value was used for DBP/MBP-calibration of radial pulse waveforms, with MBP being calculated as [0.40 × SBP + 0.60 × DBP] [35]. At least three tonometry measurements were performed, with only those having an operator index > 90% being accepted as valid, according to the quality criteria embedded in the device. Radial artery BP waveforms were then processed with pulse wave analysis. A generalized transfer function was used for the estimation of central SBP, central pulse pressure (PP), central augmentation pressure (AP), central augmentation index (AIX = AP/PP, %) and central AIX% at HR_75_ (%). The mean value of the result for each parameter was used.

### Statistical analysis

Data were analysed with IBM SPSS Statistics 27.0 (*IBM Corp., Armonk, New York, USA*). Data were checked for normality with the Shapiro–Wilk test as well as visually by Q–Q plots and histograms, and shown as mean ± SD or median [P_25_-P_75_] depending on the distribution. Pearson (r) correlations were used to examine linear associations between normally distributed continuous variables, in any other case Spearman correlation (r_s_) was used. Linear and binary logistic regression were used to investigate associations between variables of interest. Independent samples t-test and one-way ANOVA were used to compare normally distributed continuous variables between two or more independent groups, respectively. The assumption of equal variances (homoscedasticity) was evaluated with Levene’s test, and the Welch’s or Brown-Forsythe test if necessary. For comparisons of non-normally distributed variables between two or more groups, the non-parametric Mann–Whitney U test and Kruskal–Wallis test were used, respectively. Post-hoc testing with Bonferroni correction (parametric) or the Dunn's test (non-parametric) was used for multiple comparisons. Level of significance was set at p < 0.05.

## Results

### Patient characteristics

Fifty-four patients (n = 54; 32 male, 22 female), aged 46 ± 9.5 years (range: 26–68 years), with T1D duration 27 ± 8.8 years (range: 11–59 years), and without overt CVD were included (Table 1).

**Table 1:** Patient characteristics

		Mean ± SD / Median [P_25_-P_75_]	n (%)
T1D duration (years)	10–19 years	27 ± 8.8	8 (14.8)
	20–29 years		29 (53.7)
	≥ 30 years		17 (31.5)
Insulin administration	MDI	–	39 (72.2)
	CSII		15 (27.8)
Smoking	Currently (yes)	–	4 (7.1)
	History (yes)		18 (33.3)
	Pack years	11 ± 7.7	
Lipid profile (mg/dl)	TC	170 ± 26.5	–
	HDL-C	59 ± 14.0	
	LDL-C	95 ± 21.1	
	TGL	68 [54.5–90.8]	
24 h ABPM (mmHg)	24-h SBP/DBP	119/73 ± 10.7/6.2	–
	Daytime SBP/DBP	124/76 ± 11.8/6.6	
	Night-time SBP/DBP	109/64 ± 10.0/6.2	
Microvascular complications		Diabetic kidney disease (yes)	18 (33.3)
		Retinopathy (yes)	24 (44.4)

MDI: multiple daily injections; CSII: continuous subcutaneous insulin infusion

Mean HbA1c was 7.8 ± 0.83%, with ten patients (18.5%) below < 7%. Twenty-five patients (46.3%) used statins or other lipid-lowering drugs. According to 2016 and 2019 ESC/EAS Guidelines, respectively 35.2% and 11.1% of patients were on target for LDL-C. Sixteen patients (29.6%) had hypertension on 24 h ABPM. Mean SBP-dipping was 11.4 ± 5.20%, with 35.8% of patients (n = 19/53) showing the non-dipping phenomenon. Twenty-one patients used antihypertensive medication (38.9%), of whom 11 (20.4%) had hypertension as indication, 17 (31.5%) albuminuria as indication, and 7 (13.0%) of them both indications.

### Arterial stiffness (cf-PWV) and association with (cardio)vascular outcomes

**Fig. 1:**
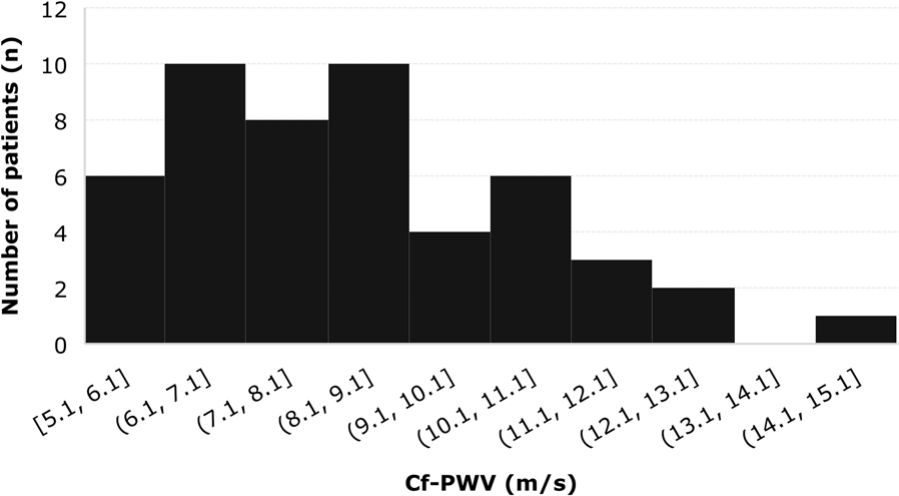
Distribution of carotid-femoral pulse wave velocity (cf-PWV; m/s) in the study population

In 50 patients, cf-PWV was measured (in two patients a reliable femoral pulse could not be obtained due to obesity; in two other patients the software failed to detect the R-wave on ECG due to presence of a left bundle branch block). Median cf-PWV was 8.3 [6.8–10.1] m/s with a range of 5.1–14.5 m/s (Fig. 1). Approximately one-fourth of the patients (n = 12/50; 24%) showed increased aortic stiffness, i.e., cf-PWV above the 90th percentile of their age- and BP-matched reference value.

Considering associations between clinical characteristics and arterial stiffness, cf-PWV showed significant associations with traditional CV risk factors age, T1DM duration, and 24-h mean arterial pressure (p < 0.01); cf-PWV was not associated with BMI or lipid parameters; and concerning glycaemic control, cf-PWV was associated with current HbA1c and mean 10-years HbA1c (p < 0.01). Multiple linear regression analysis for cf-PWV showed that the model with the best fit included age, T1DM duration, 24-h MAP and mean 10-years HbA1c (adjusted R^2^ = 0.645, p < 0.001). There were also significant associations between cf-PWV and renal parameters serum creatinine (r_s_ = + 0.36; p < 0.05), eGFR (r_s_ = − 0.47, p < 0.001) and UACR (r_s_ = + 0.39, p < 0.01), and cf-PWV was higher in patients with diabetic kidney disease versus those without (9.7 ± 2.41 vs. 7.9 ± 1.61 m/s, p < 0.01). There were no differences in cf-PWV between patients with versus without retinopathy (p = 0.165). Cf-PWV was higher in patients with hypertension compared to normotensive subjects (9.7 ± 1.67 m/s vs. 8.0 ± 2.10 m/s, p < 0.01). Lastly, cf-PWV was moderately associated with the severity of coronary calcifications as indicated by CAC score (r_s_ = + 0.34, p < 0.05), with carotid IMT (r_s_ = + 0.54, p < 0.001); and with all estimated central BP parameters (central SBP: r_s_ = + 0.58; central PP: r_s_ = + 0.48; central AP: r_s_ = + 0.62; central AIX: r_s_ = + 0.53; central AIX at HR_75_: r_s_ = + 0.58; p < 0.001). Results of the cardiovascular imaging and function outcomes (CAC score, carotid ultrasound and central hemodynamics) are listed in Table 2.

**Table 2:** Results of cardiovascular imaging and function outcomes

		Mean ± SD/Median [P_25_–P_75_]	n (%)
Coronary calcifications: calcium score (CAC)	Absolute score	No coronary calcification (0)	32 (62.7)
		Mild calcification (1–99)	14 (27.5)
		Moderate calcification (100–299)	1 (2)
		Severe calcification (≥ 300)	4 (7.8)
	Percentile score	Normal calcification (< P_50_)	34 (66.7)
		Mild calcification (P_50-75_)	6 (11.8)
		Moderate calcification (P_75-90_)	4 (7.8)
		Severe calcification (> P_90_)	7 (13.7)
Carotid artery: Presence of plaques		–	17 (31.5)
Central hemodynamic parameters	Central SBP (mmHg)	123 ± 13.4	–
	Central PP (mmHg)	47 ± 11.5	
	Central AP (mmHg)	9.0 [6.0–13.0]	
	Central AIX (%)	22 ± 10.6	
	Central AIX at HR_75_ (%)	16 ± 10.9	

### Comparison between cf-PWV, STENO score and ESC criteria for CV risk evaluation

Based on the absolute cf-PWV cut-off of > 10 m/s, 13/50 patients (26%) patients showed an increased CV event risk. According to the STENO score, the median 10-year CV event risk was 12.1% [7.4–20.1%] (ranging between 2.7 to 62.4%), with 20 patients (37%) being at low (< 10%), 20 patients (37%) at moderate (10–20%), and 14 patients (26%) at high CV event risk (≥ 20%). Using the ESC guidelines on diabetes and CVD, 10 patients (18.5%) were at high 10-year risk for a fatal CVD event (5–10%), and 44 patients (81.5%) at very high risk (> 10%).

**Fig. 2:**
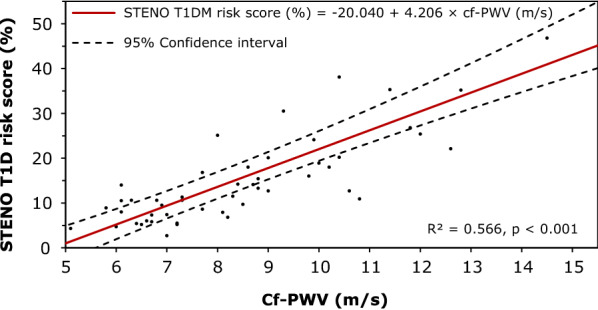
Univariate linear regression for predicting the STENO T1D CV event risk score with carotid-femoral pulse wave velocity (cf-PWV)

Cf-PWV was strongly associated with the STENO score (r_s_ = + 0.81, p < 0.001), increasing in each higher STENO group (6.9 ± 0.87, 8.4 ± 1.48 and 11.0 ± 1.77 m/s for the low-, moderate- and high-risk groups, respectively; p < 0.01). Univariate linear regression showed that cf-PWV explained almost 60% of variance in STENO score (R^2^ = 0.566, p < 0.001) (Fig. 2). Similar results were found with logistic regression, showing that for each 1 m/s increase in cf-PWV, there is a three times higher odd to be in the moderate- than in the low STENO risk group, and to be in the high- than in the moderate STENO risk group (OR = 2.912 (1.394–6.084); OR = 3.008 (1.417–6.385), respectively, p < 0.01).

**Fig. 3:**
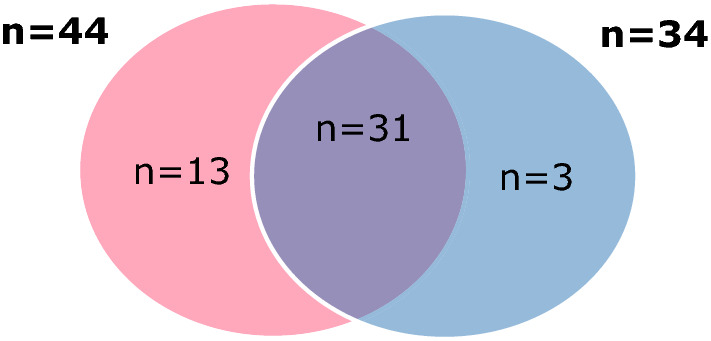
Patients with > 10% 10-year risk for CV event based on ESC criteria (red) versus STENO (blue)

Cf-PWV was not significantly different between the two CV risk groups based on ESC criteria (7.8 ± 1.56 m/s in the high-risk vs. 8.7 ± 2.20 m/s in the very high-risk group, p = 0.231). ESC criteria compared to STENO classified 10 patients more as having > 10% 10-year risk for a CV event (n = 44; 81.5% compared to n = 34; 63%) as shown in Fig. 3.

## Discussion

This study evaluated arterial stiffness in T1D patients with long disease duration and still free from known CVD and compared cf-PWV to other CV risk evaluation tools used in T1D. It was found that a substantial proportion (24%) of patients show premature arterial stiffening, which was strongly associated with the STENO risk score for future CV events. Our findings, however, also demonstrate the presence of considerable heterogeneity in arterial stiffness, STENO score and hence in CV risk, and differences in risk categorisation between the STENO tool and ESC criteria.

### Type 1 diabetes & CV risk evaluation: more precision needed

This study population with long T1D duration but still free from apparent CVD can be considered a relevant target population in CV risk assessment. However, optimal risk stratification in T1D remains unknown due to the heterogeneity in CV risk [8]. Despite the absence of clinical CVD, the cardiometabolic risk profile was suboptimal with a large proportion showing inadequate control of traditional risk factors. Only ~ 20% and ~ 10% of patients were meeting currently proposed targets for HbA1c and LDL-C, respectively. Indeed, the strict targets in T1D are often not met and hypercholesterolemia, hypertension and poor glycaemic control are still prevalent [36, 37]. Despite the high proportion of patients being above target values, less than half was using lipid-lowering or antihypertensive therapy, confirming that these risk factors are underestimated or undertreated in clinical practice [37, 38]. In particular, the timing for initiating statin therapy needs further investigation. The STENO score could assist in better assessing the CV risk profile in patients with T1D, and in determining the eligibility for statin use [11].

Considering CV risk in our study population, the wide range in STENO score (2.7–62.4%) should be noted. About 25% of patients had a high (*i.e.* ≥ 20%) 10-year risk for CV events. 37% of patients (n = 20) were still in the low STENO risk group (*i.e*., STENO < 10%) of which fifteen with a T1D duration > 20 years, while patients with a disease duration of > 20 years are generally considered at very high CVD risk (*i.e.,* 10% risk for fatal CVD) according to 2019 ESC guidelines [6]. Indeed, based on these 2019 ESC criteria, a very large proportion (81.5%) of study patients were classified as very high CVD risk, which was almost 20% more than the proportion based on STENO (63%). Our data thereby show both the heterogeneity in CV risk as well as the discrepancy between the ESC criteria and STENO score as reported before [7]. Hence, there is a need for refined CV risk classification in T1D and the development of prediction models specific for this high-risk group [9, 39]. Whereas ESC criteria—originally designed to estimate prognosis in T2D patients—are rather rigid and might overestimate CV risk in certain patients with T1D, the STENO score—considering several parameters—provides a more sophisticated evaluation of CV risk. Using STENO, discrimination for CV events was excellent in the external validation data and STENO was considered as a high-performing prediction model allowing for clinical implementation [10]. Two more recent studies showed that the STENO score identified T1D individuals with subclinical atherosclerosis and associated with the incidence of CV events [40, 41], but at the same time stated that its performance and usefulness need to be further examined in larger prospective studies. Notably, while in our study cf-PWV increased with each STENO risk group, it was not significantly different between the two ESC-based risk groups, again suggesting that STENO may be more suited for CV risk estimation than using the ‘simple’ ESC criteria.

### Early vascular aging: what’s in a name?

In our study, arterial stiffness was defined to be increased if cf-PWV was above the 90^th^ percentile of the BP- and age-matched reference value [32]. Previous research already advised BP- and age-specific PWV thresholds for vascular risk management rather than using the absolute cut-off value of 10 m/s alone, since the latter was not primarily intended to detect patients with increased arterial stiffness [42] but to identify increased CV event risk – as was derived from large longitudinal studies [22]. Still, this 10 m/s threshold was recently found to be still very valuable and accurate as a cut-off above which CV event risk is independently increased [43]. Yet, since T1D patients show *prematurely* increased arterial stiffness, comparing patients to age-related reference values allows to demonstrate the phenomenon of early vascular aging in this population. This might be important in younger T1D patients, and future studies should investigate the added value of arterial stiffness in those patients.

### Clinical importance of arterial stiffness in T1D

In our apparent CVD-free study population, a substantial proportion (24%) of patients showed prematurely increased arterial stiffness. The main clinical relevance of cf-PWV *i.e.,* its predictive value for CV events and mortality [27], was reflected in our study by the strong association (r_s_ = + 0.81) of cf-PWV with the STENO score as surrogate marker of CV risk. This is comparable to the study results from Llauradó et al*.* (r = + 0.78) also in patients free from clinical CVD [44]. Moreover, this strong association supports cf-PWV being an integrative measure of the cumulative burden of all known and unknown risk factors on the arterial wall [45], providing the opportunity to ‘*go beyond’*. Other examples in our study illustrating the clinical relevance of arterial stiffness include the significantly higher cf-PWV in patients with versus without diabetic kidney disease and the significant associations of cf-PWV with CAC score and carotid IMT, reflecting athero- and arteriosclerotic processes. The CAC score is a non-invasive estimation of coronary atherosclerotic burden and sometimes considered as a direct competitor of cf-PWV in CV risk classification [46]. Finally, our study found consistent associations between cf-PWV and central hemodynamics, of which central SBP is particularly important as this mainly determines cardiac afterload and coronary perfusion, with increased aortic SBP leading to ventricular remodelling and myocardial ischemia.

### CV reclassification potential of cf-PWV

The strong relationship between cf-PWV and STENO score was considered as a rationale for using arterial stiffness to simplify the assessment of CV risk in T1D [47]. The advantage of the STENO score is that it contains traditional CV risk factors as well as diabetes-specific factors such as T1D duration, HbA1c and albuminuria, providing a more comprehensive evaluation. Still, cf-PWV might result in even better CV risk estimation and identify patients who would be overlooked otherwise, since it reflects the *impact* of all those risk factors rather than just the *presence* of them. Studies should further investigate the role of STENO and cf-PWV in CV risk stratification in patients with T1D, *i.e.,* compare their performance in risk evaluation and evaluate whether cf-PWV shows complementary value to STENO, or if one might be preferred above the other. The abovementioned study from Llauradó et al*.* identified two cut-off points for cf-PWV (7.3 m/s and 8.7 m/s) to discriminate between moderate and high CV risk groups in T1D patients and the authors stated that these cut-offs should be further validated in larger prospective cohorts to show their value in clinical practice [44, 47]. In their study, another distance calculation method different from the gold-standard 80% of the direct distance, was used [48]. Therefore, to make validation of these cut-offs possible, values should be multiplied by 1.12 [49]. First, this would result in a ‘new’ mean cf-PWV of 8.4 instead of 7.5 m/s [44], comparable to the median cf-PWV of 8.3 m/s in our study despite the considerably longer T1D duration in our study (27 vs. 16 years). This might be due to the lower prevalence of current smokers in our cohort (only 7% versus 32%). Secondly, the 8.7 m/s cut-off for *high CV risk* would change to 9.7 m/s, which is close to the consensus threshold of 10 m/s.

### Implementation of cf-PWV assessment in clinical practice: what is next?

Our data suggest that assessment of arterial stiffness deserves specific attention in T1D. Cf-PWV might play an important role in this at-risk population by identifying those patients at higher risk for CV events. Moreover, as the predictive value of arterial stiffness for CVD was stronger in T1D subjects without previous CVD [27, 50], our study population with advanced T1D duration (90% of patients had T1D for more than 20 years) and supposedly still free from CVD, is a very relevant target group.

To implement cf-PWV as a tool for more accurate CV risk estimation in T1D clinical practice, however, more work is needed. Firstly, more long-term prospective studies need to corroborate the independent predictive value of cf-PWV in the development of CVD, as until now only one prospective study in T1D is available [27]. Secondly, if the scientific evidence is strong enough to advocate for clinical implementation of cf-PWV assessment, studies should identify in which specific patient profiles this is justified, *i.e.* which specific T1D patients benefit the most from cf-PWV measurement. Thirdly, how to make arterial stiffness ‘actionable’ is still unclear, *i.e.,* which further measures can be taken if arterial stiffness is increased. Fourth, clinical implementation of a screening method also depends on cost-effectiveness. One previous study—of small sample size and using brachial-ankle PWV—suggested that early screening could be cheap and effective among T1D patients with SBP > 130 mmHg and LDL > 102 mg/dL to identify those with elevated CV risk [51]. Lastly, the question remains when and how often arterial stiffness assessment should be repeated in case of aberrant results, and if cf-PWV-based treatment will improve hard outcomes.

### Strengths and limitations

Arterial stiffness was assessed using the gold-standard cf-PWV, according to consensus guidelines, and compared to the *Reference Values for Arterial Stiffness' Collaboration* (from > 11,000 non-diabetic people free from overt CVD [32]). The sample size was rather small and insufficient power could be a reason that we were not able to demonstrate a significant difference in cf-PWV between the ESC risk groups. Nevertheless, we were able to demonstrate a significant association between cf-PWV and STENO score*.* Additionally, the study population was well-described, with detailed information on the presence of microvascular complications as well as on markers of macrovascular disease. We included a relevant target group, being patients with long-standing T1D and without overt clinical CVD. The added value of arterial stiffness is especially present in patients without previous CVD whereas this is more debatable and probably less pronounced in patients with already established CVD. Therefore, the findings of our study cannot be applied in the latter patients.

## Conclusions

This study demonstrated that a substantial proportion of long-standing T1D patients free from known CVD show premature arterial stiffening. Cf-PWV strongly associates with the STENO risk score for future CV events and with cardiovascular imaging and function outcomes, thereby illustrating the clinical importance of arterial stiffness. The data, however, also show considerable heterogeneity in CV risk and differences in risk categorisation between the STENO tool and ESC criteria. There is a need for refinement of CV risk classification in T1D, and future studies should investigate if evaluation of arterial stiffness should be implemented in T1D clinical practice and which patients benefit the most from its assessment.

## Data Availability

The data generated and/or analysed during the current study are not publicly available due to national legislation, but are available from the corresponding author on reasonable request.

## Abbreviations

AP: Augmentation pressure
AIX: Augmentation index
ABPM: Ambulatory blood pressure monitoring
BP: Blood pressure
CAC: Calcium score
cf-PWV: Carotid-femoral pulse wave velocity
CV: Cardiovascular
CVD: Cardiovascular disease
PP: Pulse pressure
T1D: Type 1 diabetes
